# Molecular basis for the activation of thyrotropin-releasing hormone receptor

**DOI:** 10.1038/s41421-022-00477-0

**Published:** 2022-10-25

**Authors:** Su-Yu Ji, Ying-Jun Dong, Li-Nan Chen, Shao-Kun Zang, Jiawei Wang, Dan-Dan Shen, Jia Guo, Jiao Qin, Huibing Zhang, Wei-Wei Wang, Qingya Shen, Yan Zhang, Zhangfa Song, Chunyou Mao

**Affiliations:** 1 Department of Biophysics and Department of Pathology of Sir Run Run Shaw Hospital, Zhejiang University, School of Medicine, Hangzhou, Zhejiang China; 2 Center for Structural Pharmacology and Therapeutics Development, Sir Run Run Shaw Hospital, Zhejiang University School of Medicine, Hangzhou, Zhejiang China; 3 Department of General Surgery, Sir Run Run Shaw Hospital, Zhejiang University School of Medicine, Hangzhou, Zhejiang China; 4 Liangzhu Laboratory, Zhejiang University Medical Center, Hangzhou, Zhejiang China; 5 Department of Colorectal Surgery, Sir Run Run Shaw Hospital, Zhejiang University School of Medicine, Hangzhou, Zhejiang China; 6 Key Laboratory of Biological Treatment of Zhejiang Province, Hangzhou, Zhejiang China; 7 MOE Frontier Science Center for Brain Research and Brain-Machine Integration, Zhejiang University School of Medicine, Hangzhou, Zhejiang China; 8 Zhejiang Provincial Key Laboratory of Immunity and Inflammatory diseases, Hangzhou, Zhejiang China

**Keywords:** Cryoelectron microscopy, Single-molecule biophysics, Extracellular signalling molecules

Dear Editor,

Thyrotropin-releasing hormone receptor (TRHR), a class A G protein-coupled receptor (GPCR), is a key signal transducer in hypothalamus–pituitary–thyroid axis^1^. TRHR is mainly expressed in the anterior pituitary where it modulates the synthesis and release of thyroid-stimulating hormone and prolactin via mediating the actions of thyrotropin-releasing hormone (pGlu-His-Pro-NH_2_ (TRH)). Upon activation, TRHR primarily couples G_q/11_ proteins to exert its regulatory roles^2^. Here, we report the high-resolution cryo-electron microscopy (cryo-EM) structure of the TRH-bound TRHR–G_q_ signaling complex. Combined with cellular signaling assays, 3D variability analysis and molecular dynamics (MD) simulations, our results reveal the molecular basis of ligand recognition and activation of TRHR.

To improve the expression and stabilize the TRHR–G_q_ complex, we combined several strategies to assemble the complex, including an engineered construct of human TRHR with a BRIL fused to the N-terminus and the C-terminus truncated at Y348, the widely used dominant-negative Gα_q_ chimera (Gα_sq_iN, hereafter referred to as Gα_q_ for brevity) and NanoBiT tethering strategy^3,4^. The structure of TRHR–G_q_ complex was determined to a nominal global resolution of 2.7 Å by single-particle cryo-EM, allowing accurate modeling of TRH, receptor residues E13 to N336 with the exception of intracellular loop 3 (ICL3) and most residues of G_q_ (Fig. 1a; Supplementary Figs. S1–S3 and Table S1).

**Fig. 1:**
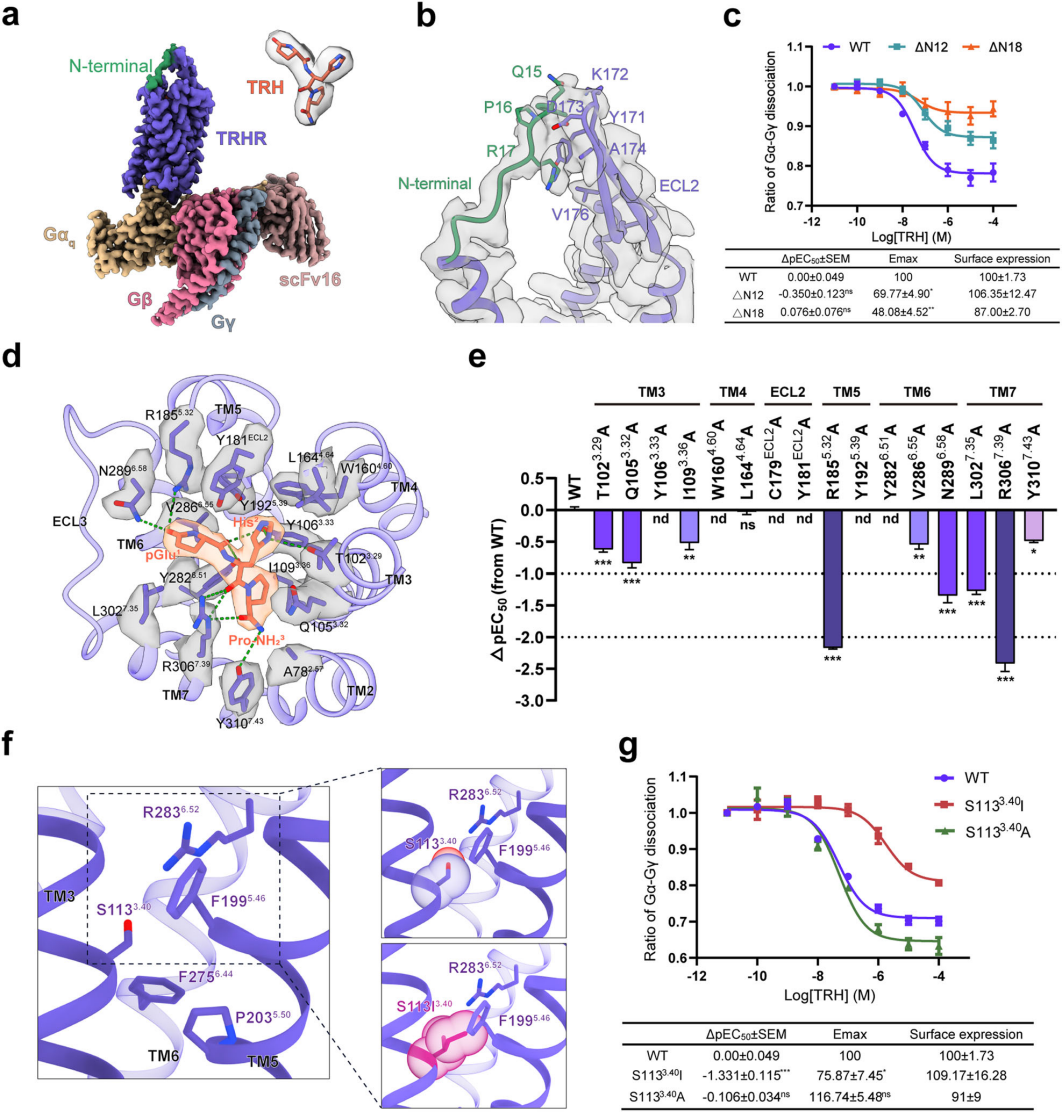
Ligand recognition and activation of human TRHR. **a** Cryo-EM structure of the TRH-bound TRHR–G_q_ complex. The density of TRH is shown. TRHR, slate blue; Gα_q_, burly wood; Gβ, pale violet red; Gγ, light slate gray; scFv16, rosy brown. The resolved N-terminus of TRHR is highlighted in sea green. **b** Interactions between the extended N-terminus and ECL2 of TRHR. **c** Effects of the N-terminal truncations (ΔN12 and ΔN18) on TRH-induced receptor activation. The G_q_ dissociation signal was detected by NanoBiT assay. **d** Detailed interactions of TRH (orange) with TRHR (slate blue). Hydrogen bonds are depicted as green dashed lines. **e** Effects of mutations in TRH-binding pocket on TRH-induced G_q_ dissociation signal as indicated by NanoBiT assay. Bars represent differences in calculated TRH potency [pEC_50_] for representative mutants relative to the wild-type receptor (WT). Data are colored according to the extent of effect. **f** Close structure examination of the non-conserved S113^3.40^ in the “PIF” motif of TRHR. Replacement of S113^3.40^ with I^3.40^ may form steric hindrance with the adjacent residues R283^6.52^ or F199^5.46^. **g** The effect of S113^3.40^ mutations in TRHR on TRH-induced G_q_ dissociation signal. Data are shown as means ± SEM from at least three independent experiments performed in technical triplicate. The cell surface expression and *E*_max_ values were normalized to WT TRHR. nd not determined, ns no significance. *P* > 0.05; *P < 0.05; **P < 0. 01; ***P < 0.001 by one-way ANOVA followed by Dunnett’s multiple comparisons test, compared with WT.

Compared with recently reported two structures of TRH-bound TRHR–G_q_ complexes^5,6^, our higher-resolution reconstruction provides a more accurate template to characterize the peptide recognition and activation of TRHR. Of note, our structure resolved an extended N-terminal region (residues 13–21) of the receptor, which has not been observed in both previously solved structures^5,6^ (Fig. 1a, b). The N-terminal portion points towards the extracellular loop 2 (ECL2) of the receptor and makes extensive contacts with the residues 171–176 at the tip of the conserved β-hairpin (Fig. 1b). The extreme N-terminus of TRHR (residues 1–12) was not resolved in our cryo-EM map, likely owing to its intrinsic flexibility. However, truncation of the N-terminal twelve residues (residues 1–12; ΔN12) appeared to compromise the activation of TRHR (Fig. 1c; Supplementary Tables S2 and S3). Further deletion of the N-terminal residues that contact ECL2 (residues 1–18; ΔN18) led to a substantial 50% reduction in maximal responses (*E*_max_) of TRH (Fig. 1c; Supplementary Tables S2 and S3). Nevertheless, our MD simulation analysis indicated that both the ΔN12 and ΔN18 mutants did not jeopardize the binding of the agonist, exhibiting a similar and marginal root mean square deviation (RMSD) value of ~0.9 Å for TRH (Supplementary Fig. S4). These results suggest that the N-terminal portion of TRHR may allosterically regulate the activation of TRHR.

TRH occupies a canonical ligand pocket in the seven-transmembrane (7TM) bundle, with its C-terminal Pro-NH_2_ located in the receptor core and the N-terminal pGlu pointing towards ECL3 (Fig. 1d). Compared with other class A peptide GPCR complexes solved so far, TRH sits into the 7TM core as deeply as most of the class A peptide agonists, except for neurotensin and galanin that bind superficially to ligand pocket^7,8^ (Supplementary Fig. S5). However, the tripeptide TRH forms much fewer interactions with the extracellular end of 7TM and ECLs, displaying a smaller interface (522 Å^2^) with the receptor than that of other peptide agonists (Fig. 1d; Supplementary Fig. S5). Detailed interaction analysis revealed that TRH forms extensive hydrogen-bonding or polar interactions with TRHR, involving the residues in TM3/5/6/7 and ECL2 (T102^3.29^, Y106^3.33^, Y181^ECL2^, R185^5.32^, Y192^5.39^, Y282^6.51^, N289^6.58^, R306^7.39^, Y310^7.43^, superscripts refer to Ballesteros–Weinstein numbering^9^) (Fig. 1d; Supplementary Table S4). Our cellular signaling assay showed that most alanine mutations severely compromised TRH activity (Fig. 1e; Supplementary Fig. S6 and Tables S2 and S3). Most strikingly, mutations of a succession of tyrosine residues (Y106^3.33^, Y181^ECL2^, Y192^5.39^, Y282^6.51^) abolished the downstream signals, highlighting their important roles in TRH binding and receptor activation (Fig. 1e; Supplementary Fig. S6b–d and Tables S2 and S3). In addition, our functional assay also illustrated that the residue W160^4.60^, which packs strongly against the His^2^ of TRH and the tyrosine patch (Y106^3.33^, Y181^ECL2^ and Y192^5.39^), is of great importance to TRH activity (Fig. 1d, e; Supplementary Fig. S6c and Tables S2 and S3). Compared with recently solved structures^5,6^, our structure defined a more accurate ligand-binding pose and detailed interactions (Fig. 1d; Supplementary Table S4). Specifically, owing to limited resolution, the densities of the carboxamide group of TRH were not observed in other reported maps and were modeled differently in the corresponding structures^5,6^. Our high-quality cryo-EM map is clear enough to model the carboxamide group in an “up” configuration, providing a more precise template for further drug discovery (Fig. 1d; Supplementary Fig. S5a).

The resolved TRHR structure adopts a classic active-state conformation of class A peptide GPCRs, highly similar to the reported cholecystokinin A receptor (CCK_A_R) with a Cα RMSD value < 1 Å for the 7TM bundle^10^ (Supplementary Fig. S7a). Meanwhile, the conserved “micro-switches” (Toggle switch, DRY, NPxxY, PIF motif) that are essential for the activation of class A GPCRs show almost identical conformations between TRHR and CCK_A_R, suggesting a conserved activation mechanism for TRHR^11^ (Supplementary Fig. S7b). Intriguingly, the conserved I^3.40^ in the P^5.50^I^3.40^F^6.44^ motif of class A GPCRs is replaced by a rare S113^3.40^ in TRHR and a T^3.40^ in CCK_A_R, respectively (Fig. 1f; Supplementary Fig. S7c). Structural analysis indicated that substitution of S113^3.40^ with the typical I^3.40^ would cause steric hindrance with the adjacent residues R283^6.52^ or F199^5.46^ of TRHR, which might impair the receptor activation (Fig. 1f). As expected, replacement of S113^3.40^ with I^3.40^ markedly compromised the TRH-induced receptor activation, whereas the S113^3.40^A mutation with no disruption to the local residue conformations retained the comparable signaling as the wild-type receptor (Fig. 1f, g; Supplementary Tables S2 and S3). Consistently, sequence alignment of TRHR from different species demonstrated that the functional residues S^3.40^ and A^3.40^ but not I^3.40^ are highly preserved in TRHR orthologs (Supplementary Fig. S8). Unlike TRHR, the substitution of T129^3.40^ with I^3.40^ in CCK_A_R seemed to enhance the hydrophobic interactions with V125^3.36^, L217^5.46^ and F330^6.52^ (Supplementary Fig. S7c). Indeed, our cellular signaling assays showed that mutation of T129^3.40^ with I^3.40^ in CCK_A_R evidently increased the agonist potency (Supplementary Fig. S7d). These results highlight the commonality and diversity of the activation mechanisms among class A GPCRs.

The TRHR–G_q_ interface involves TM2/3/5/6/7, ICL1/2 and helix 8 of the receptor and α5-helix, β1, αN-helix and Gβ of G_q_, with a total interface area of 1483 Å^2^ (Supplementary Fig. S9a, b). Structural comparisons of the reported GPCR–G_q_ complexes showed that G_q_ inserted into a similar cavity formed by the intracellular ends of TMs and ICLs, but rotated within a range of 15° (Supplementary Fig. S9c–e). To get insights into the structural dynamics between TRHR and G_q_, we further performed 3D variability analysis using the final particles for 3D reconstruction. 3D variability analysis revealed that the overall conformation of the TRHR–G_q_ complex is stable, with only slight motions observed in the N-terminus (~2.5 Å), ECL2 (~3.0 Å) and helix 8 (2.7 Å) of the receptor, the TRH agonist (~0.8 Å) and the coupled G_q_ (~1.2 Å) for both components (Supplementary Fig. S10).

In conclusion, we report the high-resolution cryo-EM structure of the TRH-bound TRHR–G_q_ complex, which provides molecular insights into TRHR activation. Compared with the recent two related studies^5,6^, our work provides additional structural and functional details. First, our structure resolved an extended N-terminal region (residues 13–21) of TRHR, which may allosterically regulate the receptor activation (Fig. 1a–c). Second, our higher-resolution structure defined a more accurate ligand-binding pose and interactions providing a precise platform for drug discovery (Fig. 1d, e; Supplementary Table S4). Third, our results revealed that the non-conserved S^3.40^ or A^3.40^ in the PIF motif is critical for TRHR activation and is highly preserved in TRHR orthologs (Fig. 1f, g; Supplementary Fig. S8). Collectively, these findings, together with recent studies^5,6^, uncover the molecular mechanisms for the TRH binding and activation of TRHR.

## Supplementary information

Supplementary Information

## Data Availability

The atomic coordinate and the electron microscopy map of the TRH-bound TRHR–G_q_ complex have been deposited in the Protein Data Bank (PDB) under accession number 7XW9 and Electron Microscopy Data Bank (EMDB) under accession code EMD-33494, respectively.
