# Supplementary material for: Molecular basis for the activation of thyrotropin-releasing hormone receptor
Source: Cell Discov. 2022 Oct 25;8:116. doi: 10.1038/s41421-022-00477-0 (PMC9592576; doi:10.1038/s41421-022-00477-0)
Supplement: Supplementary file 1 — Supplementary Information [file 41421_2022_477_MOESM1_ESM.pdf]

Materials and methods

Constructs

For protein purification, *human* TRHR (UniProt: P34981) were cloned into a modified pFastBac1 vector (Invitrogen) with an N-terminal hemagglutinin signal sequence connected to a Flag-tag and a C-terminal TEV protease cleavage site followed by LgBit and double MBP tag. To further improve TRHR expression, a thermostabilized BRIL was fused to the N-terminus and the C terminus was truncated at Y348. The $G\alpha_q$ chimera was designed based on a Gαs skeleton with N-terminus replaced by G_{i1} for the binding of scFv16[1]. *Human* $G\beta_1$ was fused with an N-terminal 6×His tag and a C-terminal HiBiT, together with $G\gamma_2$ were cloned into pFastBac dual vector. The scFv16 gene was cloned into the pFastBac1 with an N-terminal GP67 signaling peptide and a C-terminal 8× His-tag. For cellular signaling assays, the N-terminal Bril-fusion TRHR were sub-cloned into the pcDNA3.1 with the addition of an N-terminal Flag tag for cell-surface ELISA assay. The TRHR mutants were generated using site-directed mutagenesis. All the constructs were confirmed by sequencing.

Expression and purification of scFv16

The expression and purification of scFv16 were achieved as previously described[2]. In brief, the 6×histidine-tagged scFv16 was expressed in secreted form in *Trichoplusia ni* Hi5 insect cells for 48 h using Bac-to-Bac system. The expressed scFv16 was purified using a Ni-NTA resin. The C-terminal 6×His-tag of scFv16 was cleaved by 3C protease and further purified by gel filtration chromatography using a Superdex 200 column. Finally, the purified scFv16 was concentrated, flash frozen and stored at -80 °C until further use.

Expression and purification of TRHR-Gq complex

High titre recombinant baculoviruses were generated using Bac-to-Bac Baculovirus Expression System (Invitrogen). *Spodoptera frugiperda* (Sf9) insect cells were infected with viruses encoding TRHR, $G\alpha_q$ and $G\beta_1\gamma_2$ at equal multiplicity of infection. Cells

were cultured at 27 °C, 130 rpm for 48 h. Then, the cells were collected and stored at -80 °C until use. The cell pellets were thawed in 20 mM HEPES pH 7.5, 100 mM NaCl, 2 mM $MgCl_2$, 2 mM $CaCl_2$ supplemented with Protease Inhibitor Cocktail (Bimake) by dounce homogenization. Complex formation was initiated by adding 2 mg scFv16, 25 mU/mL apyrase (Sigma) and 100 μM TRH and further incubated for 1 h at room temperature (RT). The membrane was solubilized using 0.5% (w/v) lauryl maltose neopentyl glycol (LMNG, Anatrace) and 0.1% (w/v) cholesterol hemisuccinate (CHS, Anatrace) for 2 h at 4 °C. The sample was clarified by centrifugation at 30,000× g for 30 min and the supernatant was then incubated with amylose resin for 1 h at 4 °C. The resin was collected and washed with 15 column volumes (CVs) of 20 mM HEPES, pH 7.5, 100 mM NaCl, 2 mM $MgCl_2$, 100 μM TRH, 0.01% (w/v) LMNG and 0.005% (w/v) CHS. The complex was eluted with the buffer containing 10 mM maltose and incubated with TEV protease for 1 h at RT. Finally, the sample was concentrated using a 100 kDa cut-off concentrator (Millipore) and loaded onto a Superose 6 increase 10/300 GL column (GE Healthcare) pre-equilibrated with size buffer containing 20 mM HEPES, pH 7.5, 100 mM NaCl, 2 mM $MgCl_2$, 100 μM TRH, 0.00075% (w/v) LMNG, 0.0002% (w/v) CHS and 0.00025% (w/v) GDN (Anatrace). The fractions for the monomeric complex were collected and concentrated for electron microscopy experiments.

Cryo-EM grid preparation and data collection

The purified TRHR-Gq complex (3 μl) were applied onto the glow-discharged grids (Quantifoil, R1.2/1.3, 300 mesh) at a concentration of 18 mg ml^{-1}. The grids were blotted for 3.0 s with a blot force of 3 at 4 °C, 100% humidity, and then plunge-frozen in liquid ethane using Vitrobot Mark IV (Thermo Fischer Scientific). Cryo-EM data collection was performed on a Titan Krios at 300 kV accelerating voltage in the Center of Cryo-Electron Microscopy (Zhejiang University). Micrographs were recorded using a Gatan K2 Summit Detector in super-resolution mode with a pixel size of 1.014 Å using SerialEM software[3]. Image stacks were obtained at a dose rate of 8.0 electrons per $Å^2$ per second with a defocus ranging from -1.0 to -2.5 μm. The total exposure time

was 8 s, and 40 frames were recorded per micrograph. A total of 3,667 movies were collected for the TRHR-Gq complex.

Cryo-EM data processing

Image stacks were aligned using MotionCor 2.1[4]. Contrast transfer function (CTF) parameters were estimated by Gctf v1.18[5]. The following data processing was performed using RELION 3.1 and CryoSPARC v3.1[6,7]. Automated particle selection using Gaussian blob detection in RELION 3.1 produced 3,687,308 particles. The particles were imported to CryoSPARC v3.1 for two rounds of heterogeneous refinement using the map of GAL2R–Gi complex (EMD-32698)[8] as the initial reference. The well-defined subsets accounting for 2,342,376 particles were re-extracted and subject to 3D classifications in RELION 3.1, resulting in two well-defined subsets with 1,172,177 particles. To further improve the map quality, another round of 3D classification focusing the alignment on the complex without detergent micelle produced one high-quality subset accounting for 324,020 particles, which were subsequently subjected to 3D refinement, CTF refinement and Bayesian polishing. The final refinement generated a map with an indicated global resolution of 2.7 Å at a Fourier shell correlation of 0.143. The final map of TRH-bound complex was sharpened with deepEMhancer[9] and used for subsequent model building and analysis. Local resolution was determined using the Bsoft package[10] with half maps as input maps. 3D variability analysis of TRHR-Gq complex was performed in cryoSPARC with 2 components. Then, the model of TRHR-Gq complex was subjected to flexible fitting into two maps (frame000 and frame019) of component 1 and 2 using Rosetta 2019.35[11].

Model building and refinement

The AlphaFold2 predicted model of TRHR was used to generate the initial template of receptor[12]. The atomic coordinates of G_q and scFv16 from the structures of Gq- and Gi-coupled galanin receptors (PDB: 7WQ3 and 7WQ4)[8] were used to generate the initial template of the G protein complex. Models were docked into the density map using

UCSF Chimera[13]. Agonists coordinates and geometry restraints were generated using a phenix.elbow[14]. The docked model was subjected to flexible fitting using Rosetta 2019.35[11] and iterative manual adjustment in Coot[11] and real-space-refined in Phenix[14]. The final refinement statistics were validated using the module 'comprehensive validation (cryo-EM)' in Phenix. The goodness-of-fit of the model to the map was determined using a global model-versus-map Fourier shell correlation. The refinement statistics are provided in Supplementary Information, Table S1. Structural figures were created using UCSF Chimera[13] and the UCSF Chimera X package[15].

NanoBiT G-protein dissociation assay

The recruitment of TRHR to Gq was detected in HEK293T cells using NanoBiT Systems (Promega)[16]. *Human* TRHR-LgBiT, WT-Gαq, SmBiT-$G\beta_1$ and $G\gamma_2$ were co-transfected in HEK293T cells in the 6-well plate using the Hieff Trans™ Liposomal Transfection Reagent (YEASEN). After one day of transfection, cells were seeded in 96-well flat-bottomed white microplates in a total volume of 100 μl per well. After 24 hours, the cells were washed twice with D-PBS and incubated in 40 μl of 5 μM coelenterazine 400a (Promega) solution diluted with HBSS assay buffer containing 0.01% BSA and 5 mM HEPES pH 7.4 for 35 min at RT. Baseline luminescence was measured 5 cycles using a luminescent microplate reader (Tecan). The ligand TRH (5×, diluted in HBSS assay buffer) was added to the cells (10 μl) and incubated for 3-5 min at RT before the second measurement. The ligand-induced signal ratio was normalized to the baseline luminescence, and fold-change signals over vehicle treatment were used to show the G-protein dissociation response.

cAMP accumulation assay

CCK8-stimulated cAMP accumulation was measured by a GloSensor™ cAMP assay kit (Promega). Briefly, HEK293 cells were transfected with a plasmid mixture consisting of Flag-CCK_AR and the cAMP biosensor GloSensor-22F (Promega) at a ratio of 2:1. After incubation at 37 °C for 24 h, transfected cells were seeded into 96-

well plates and incubated for another 12 h. Cells were treated with Hank's balanced salt solution for starvation and then incubated in CO_2-independent media containing 2% GloSensor cAMP Reagent (Promega) at a volume of 50 µl per well before measurements for baseline luminescence (Spark Multimode microplate reader, TECAN). Next, CCK8 was added at different concentrations from 10^{-4} to 10^{-11} M. All luminescence values were first normalized by the initial counts before ligand treatments. Fold-change signals over the treatment of the lowest CCK8 concentration were used to show intracellular cAMP response. Data were analysed using the sigmoidal dose-response function in GraphPad Prism 8.0.

Cell-surface ELISA

The cell-surface expression of TRHR was detected using an enzyme-linked immunosorbent assay (ELISA). HEK293T cells were seeded and transiently transfected with WT or mutant Flag-TRHR using Hieff Trans™ Liposomal Transfection Reagent (YEASEN) in 200 µl of Opti-MEM (Thermo Fisher Scientific). After one day of transfection, cell was plated in a 96-well plate in a total volume of 100 µl per well and were incubated for another 24 h at 37 °C in 5% CO_2. HEK293T cells were fixed with 4% paraformaldehyde for 10 min and then blocked with 1% BSA (Bovine Serum Albumin) for at least 1 h at room temperature. Bound antibodies coupled to horseradish peroxidase were detected by luminescence using SuperSignal ELISA Femto Maximum Sensitivity substrate (ThermoFisher Scientific), and luminescence was measured using a luminescence microplate reader (Tecan).

Molecular dynamics simulations

The models of TRH-bound TRHR truncations (ΔN12 and ΔN18) were generated from our cryo-EM structure. The orientations of TRHR are calculated by the Orientations of Proteins in Membranes database. Then, the whole systems were prepared by the CHARM-GUI and embedded in a bilayer consisting of 100 1-palmitoyl-2-oleoyl-sn-glycero-3-phosphocholine lipids by replacement methods. The membrane systems

were then solvated into a periodic TIP3P water box supplemented with 0.15 M NaCl. The CHARMM36m Force Field was used to model protein molecules. Then the systems were subjected to minimization for 10,000 steps using the conjugated gradient algorithm, and then heated and equilibrated at 310.13 K and 1 atm for 200 ps with 10.0 kcal mol^{-1} $Å^{-2}$ harmonic restraints in the NAMD v.3.0. After that, there were five cycles of equilibration for 2 ns each at 310.13 K and 1 atm, at which the harmonic restraints were 5.0, 2.5, 1.0, 0.5 and 0.1 kcal mol^{-1} $Å^{-2}$ in sequence. Production simulations were run at 310.13 K and 1 atm in the NPT ensemble using the Langevin thermostat and Nose–Hoover method for 500 ns. Electrostatic interactions were calculated using the particle mesh Ewald method with a cutoff of 12 Å. Throughout the final stages of equilibration and production, 5.0 kcal mol^{-1} $Å^{-2}$ harmonic restraints were placed on the residues of TRHR that were within 4 Å of Gq in our cryo-EM structure to ensure that the receptor remained in the active state without G protein. Trajectories were visualized and analysed using Visual Molecular Dynamics (VMD, v1.9.3).

Statistical analysis

Statistical analyses were performed on at least three individual datasets and analysed using GraphPad Prism software. Bars represent differences in the calculated agonist potency (pEC_{50}) and maximum agonist response (E_{max}) for each mutant relative to the wild-type receptor. Data are mean ± S.E.M. from at least three independent experiments, performed in triplicates. nd, not determined; ns, not significant, $^{ns}P > 0.05$; $*P < 0.05$; $**P < 0.01$; $***P < 0.001$ (one-way analysis of variance (ANOVA) followed by Dunnett's test, compared with the response of the wild type). For dose-response experiments, data were normalized and analysed using nonlinear curve fitting for the log (agonist) versus response (three parameters) curves.

Reference

1 Mobbs, J. I. *et al.* Structures of the human cholecystokinin 1 (CCK1) receptor bound to Gs and Gq mimetic proteins provide insight into mechanisms of G protein selectivity. *PLoS Biol* **19**, e3001295, doi:10.1371/journal.pbio.3001295 (2021).

2 Koehl, A. *et al.* Structure of the micro-opioid receptor-Gi protein complex. *Nature* **558**, 547-552, doi:10.1038/s41586-018-0219-7 (2018).

3 Schorb, M., Haberbosch, I., Hagen, W. J. H., Schwab, Y. & Mastronarde, D. N. Software tools for automated transmission electron microscopy. *Nat Methods* **16**, 471-477, doi:10.1038/s41592-019-0396-9 (2019).

4 Zheng, S. Q. *et al.* MotionCor2: anisotropic correction of beam-induced motion for improved cryo-electron microscopy. *Nat Methods* **14**, 331-332, doi:10.1038/nmeth.4193 (2017).

5 Zhang, K. Gctf: Real-time CTF determination and correction. *J Struct Biol* **193**, 1-12, doi:10.1016/j.jsb.2015.11.003 (2016).

6 Scheres, S. H. Processing of Structurally Heterogeneous Cryo-EM Data in RELION. *Methods Enzymol* **579**, 125-157, doi:10.1016/bs.mie.2016.04.012 (2016).

7 Punjani, A., Rubinstein, J. L., Fleet, D. J. & Brubaker, M. A. cryoSPARC: algorithms for rapid unsupervised cryo-EM structure determination. *Nat Methods* **14**, 290-296, doi:10.1038/nmeth.4169 (2017).

8 Duan, J. *et al.* Molecular basis for allosteric agonism and G protein subtype selectivity of galanin receptors. *Nature communications* **13**, 1364, doi:10.1038/s41467-022-29072-3 (2022).

9 Sanchez-Garcia, R. *et al.* DeepEMhancer: a deep learning solution for cryo-EM volume post-processing. *Commun Biol* **4**, 874, doi:10.1038/s42003-021-02399-1 (2021).

10 Heymann, J. B. Single particle reconstruction and validation using Bsoft for the map challenge. *Journal of Structural Biology* **204**, 90-95, doi:10.1016/j.jsb.2018.07.003 (2018).

11 Emsley, P. & Cowtan, K. Coot: model-building tools for molecular graphics. *Acta crystallographica. Section D, Biological crystallography* **60**, 2126-2132, doi:10.1107/s0907444904019158 (2004).

12 Jumper, J. *et al.* Highly accurate protein structure prediction with AlphaFold. *Nature* **596**, 583-589, doi:10.1038/s41586-021-03819-2 (2021).

13 Pettersen, E. F. *et al.* UCSF Chimera--a visualization system for exploratory research and analysis. *J Comput Chem* **25**, 1605-1612, doi:10.1002/jcc.20084 (2004).

14 Adams, P. D. *et al.* PHENIX: a comprehensive Python-based system for macromolecular structure solution. *Acta crystallographica. Section D, Biological crystallography* **66**, 213-221, doi:10.1107/s0907444909052925 (2010).

15 Goddard, T. D. *et al.* UCSF ChimeraX: Meeting modern challenges in visualization and analysis. *Protein science : a publication of the Protein Society* **27**, 14-25, doi:10.1002/pro.3235 (2018).

16 Inoue, A. *et al.* Illuminating G-Protein-Coupling Selectivity of GPCRs. *Cell* **177**, 1933-1947.e1925, doi:10.1016/j.cell.2019.04.044 (2019).

Supplementary Fig. S1

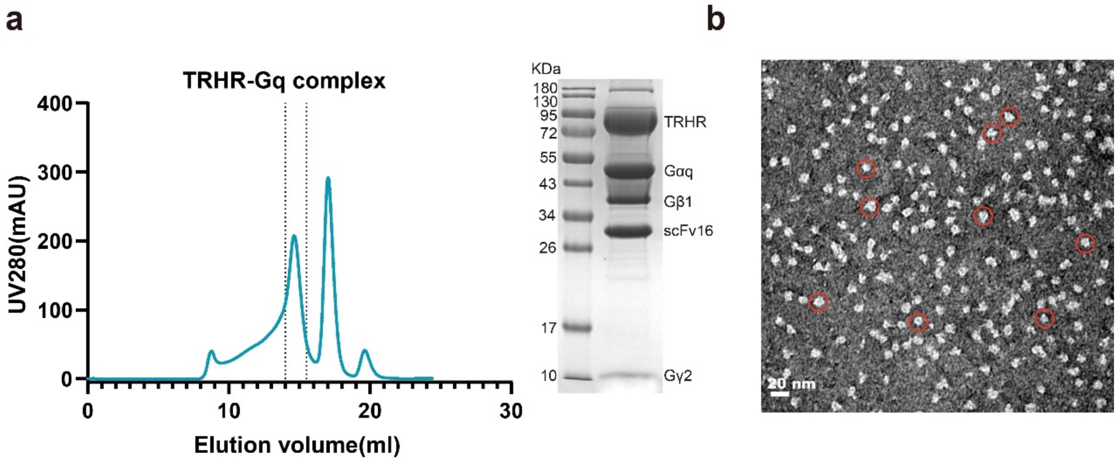

Fig. S1 Purification of the TRH-bound TRHR-Gq complex.

a Size exclusion chromatography (SEC) profile and SDS-PAGE analysis of TRH-bound TRHR-Gq complex. **b** Negative stain analysis of the pooled fractions between two dashed lines in the SEC profile (scale bar: 20 nm). Examples of TRHR-Gq complex are circled in red.

Supplementary Fig. S2

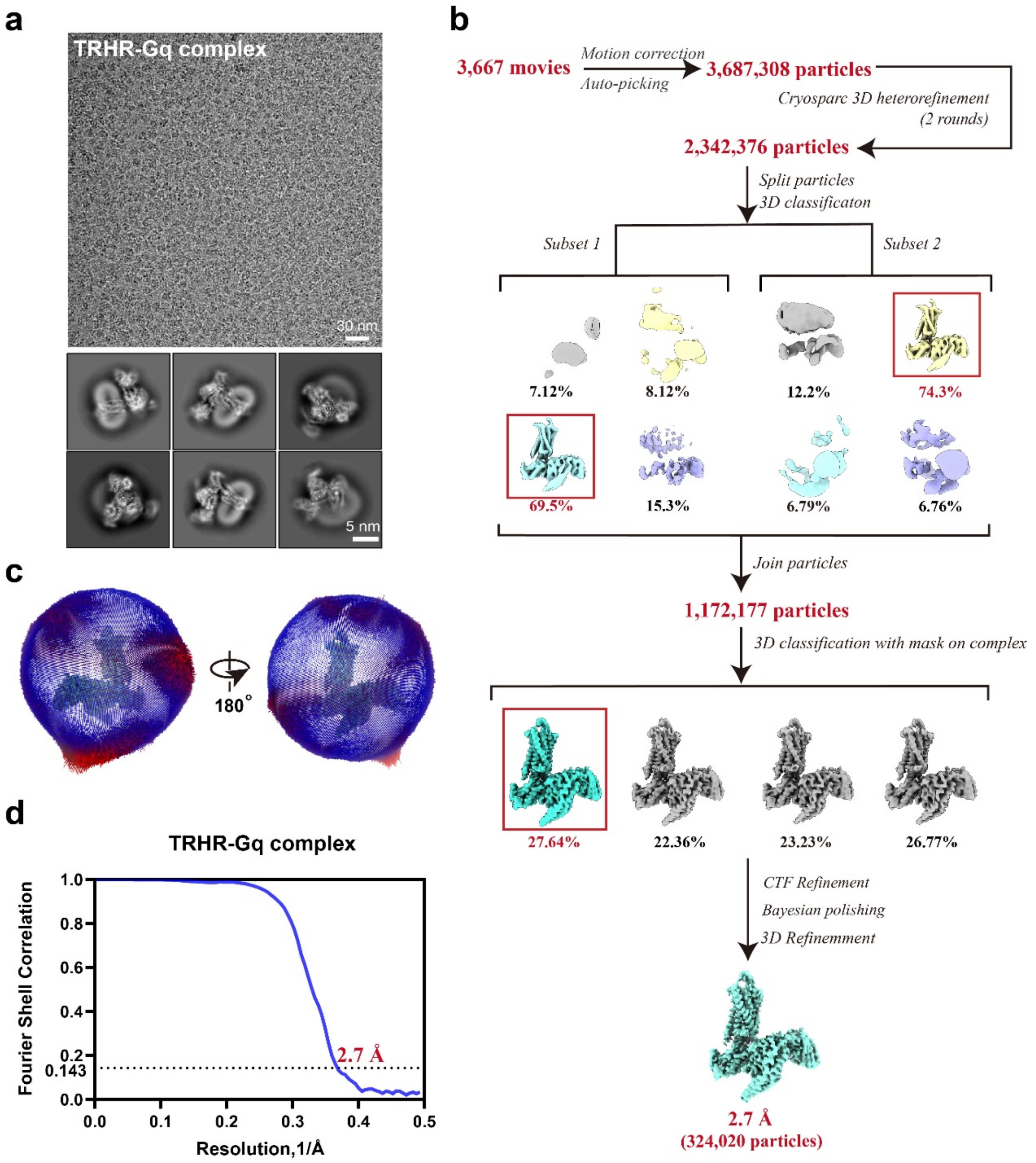

Fig. S2 Cryo-EM data processing of the TRHR-Gq complex.

a Cryo-EM micrograph (scale bar: 30 nm) and 2D class averages (scale bar: 5 nm) of TRHR-Gq complex. **b** Flow chart of cryo-EM data processing. **c** Angular distribution of particles used in the final 3D reconstruction. **d** Gold-standard Fourier shell correlation (FSC) curve of the final refined map.

Supplementary Fig. S3

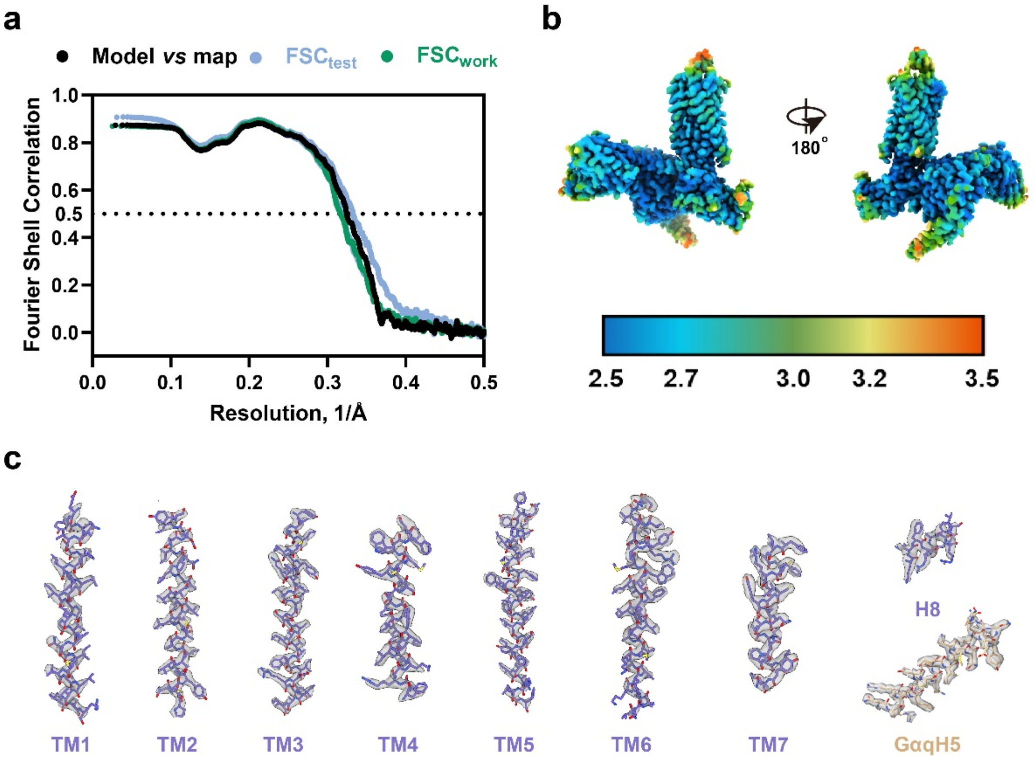

Fig. S3 Local resolution and cryo-EM density analysis.

a Fourier shell correlation curves of the model-vs-map, FSC_{work} and FSC_{test}. The marginal gap between the FSC_{work} and FSC_{test} curves indicate no over-fitting of the model. **b** Cryo-EM maps coloured by local resolution. **c** Cryo-EM density maps and models are shown for all seven-transmembrane helices, helix 8, Gαq α5-helix of the TRHR-Gq complex.

Supplementary Fig. S4

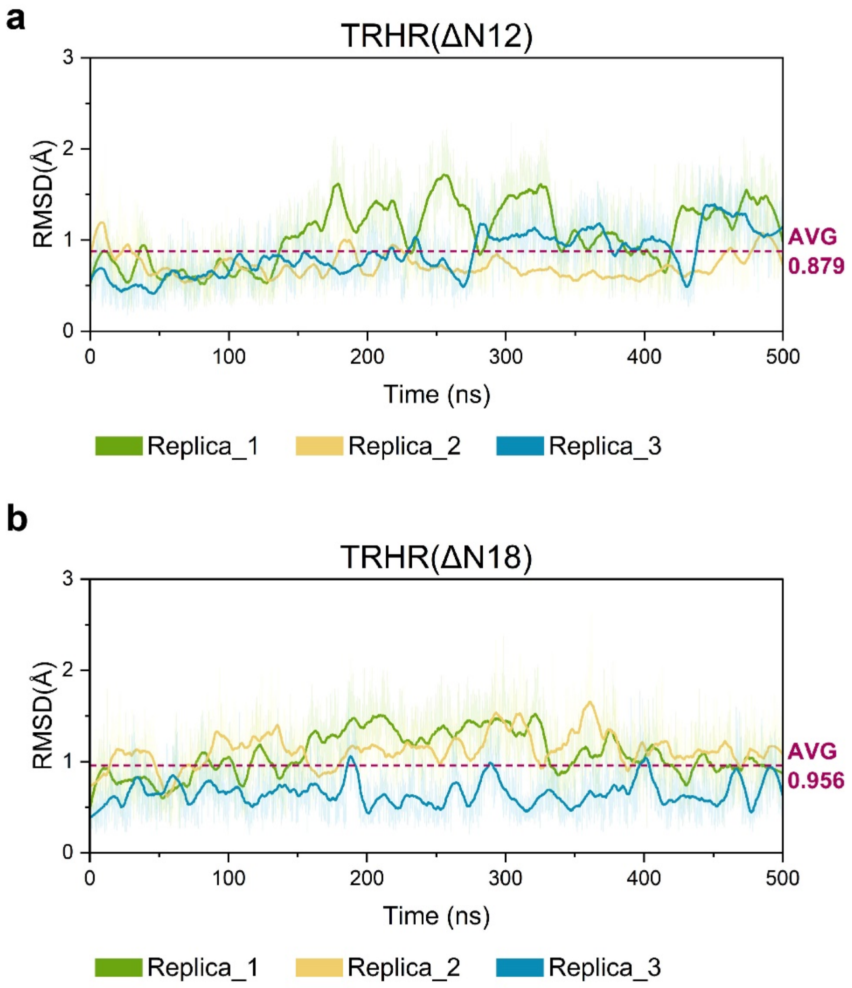

Fig. S4 Molecular dynamics simulations of the TRH-bound TRHR with and without the N-terminal portion that engages the ECL2.

a-b RMSD analysis of the TRH binding with (ΔN12) and without(ΔN18) the N-terminal portion of TRHR during 500 ns MD simulation. Simulations were run over 500 ns and 3 simulations per condition.

Supplementary Fig. S5

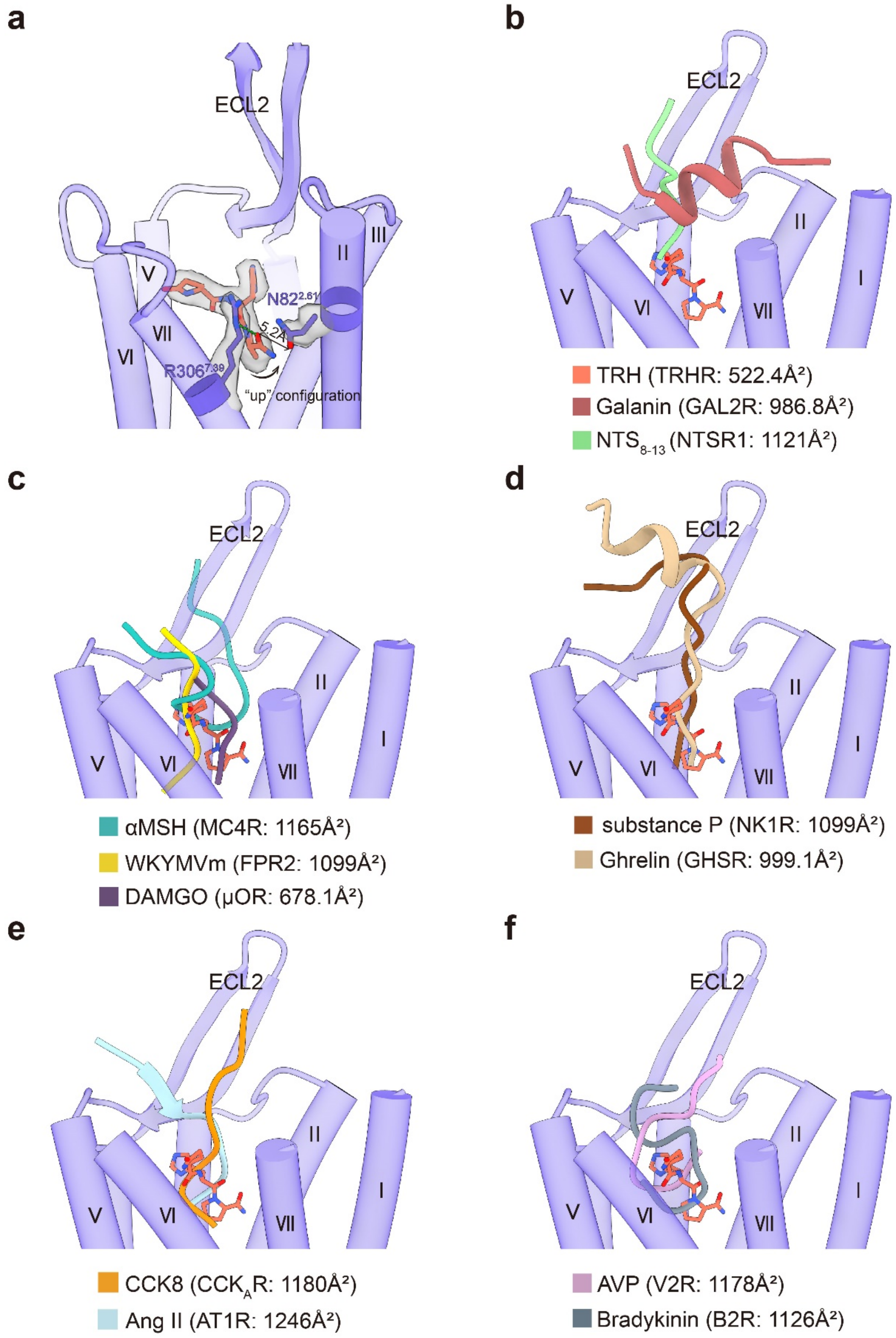

Fig. S5 The TRH-binding mode of TRHR.

a Our high-resolution cryo-EM map defined accurate TRH-binding pose and surrounding interactions. The carboxamide group forms hydrogen-bonds with NH

group of R306$^{7.39}$ but not that with N82$^{2.61}$ (distance > 5 Å) that described in the recent study *(Xu, Y. et al. Cell Res. 2022)* with the same "up" configuration. Hydrogen bonds are depicted as green dashed lines. **b-f** Structural comparisons of the peptide-binding mode between TRHR and other class A peptide receptors including galanin receptor 2 (GAL2R, PDB: 7WQ4); neurotensin receptor 1 (NTSR1, PDB: 7L0P); melanocortin 4 receptor (MC4R, PDB: 7F53); formyl peptide receptor 2 (FPR2, PDB: 6OMM); mu-opioid receptor (μOR, PDB: 6DDE); cholecystokinin A receptor (CCK$_A$R, PDB: 7EZM); neurokinin 1 receptor (NK1R, PDB: 7P00); ghrelin receptor (GHSR, PDB: 7F9Y); vasopressin receptor 2 (V2R, PDB: 7DW9); bradykinin receptor type 2 (B2R, PDB: 7F2O); angiotensin II receptor type 1 (AT1R, PDB: 6OS0). Structures were aligned by the receptors; only TRHR is shown for clarity. The interface area between the peptide agonists and the corresponding receptors were measured by Chimera 1.15.

Supplementary Fig. S6

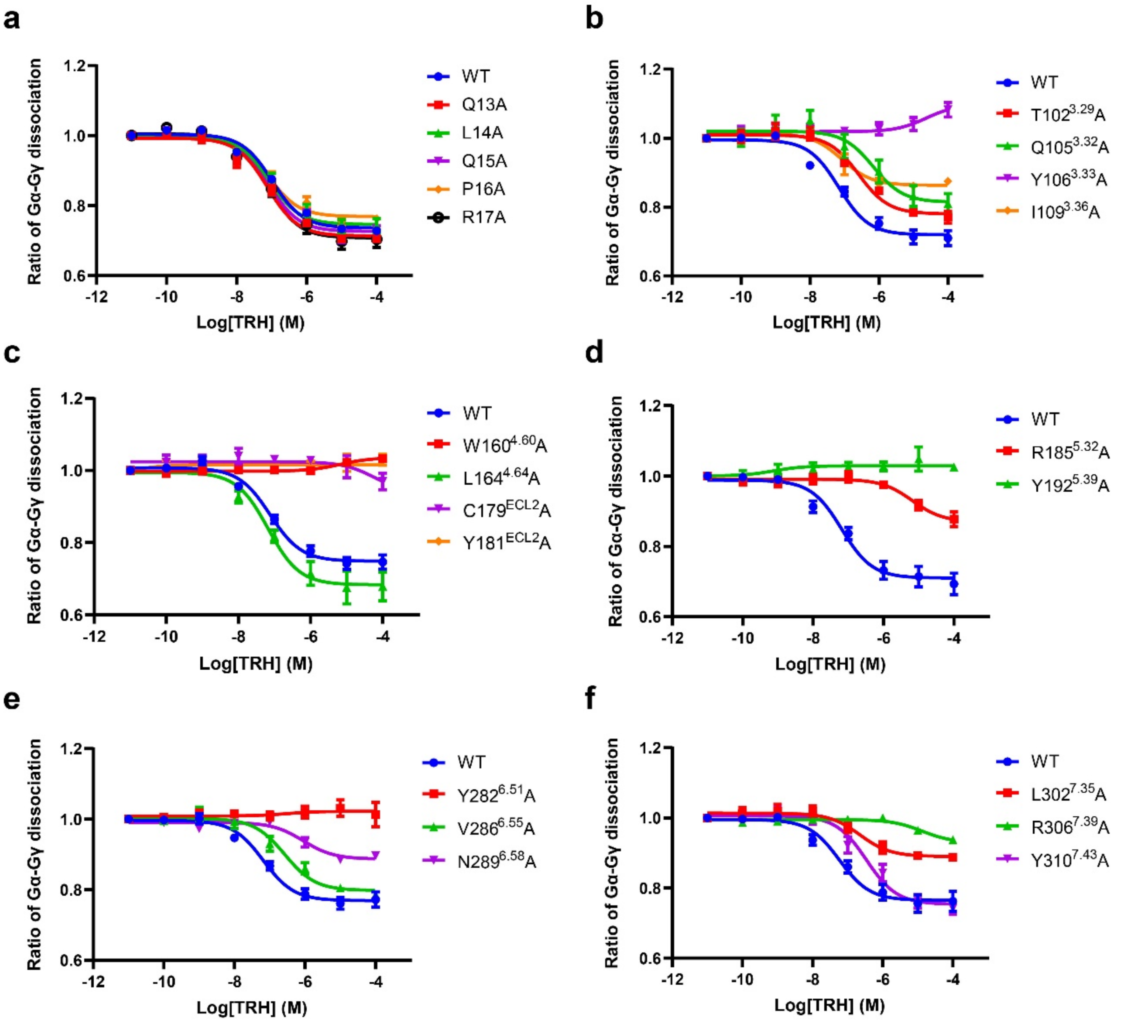

Fig. S6 Response curves of G-protein dissociation assay on the N-terminal and TRH-binding pocket of TRHR.

a-f Dose-response curves of TRHR variants harboring mutations in the N-terminal portion and TRH-binding pocket. Disruption of the conserved disulfide bond (C179^{ECL2}A) crucial for the stability of ECL2 completely damaged the ligand-induced activation. This result suggests that ECL2 plays a vital role in the peptide binding of TRHR, which is similar to other class A peptide receptors (**c**). The G-protein dissociation signal was detected by NanoBiT assay. Data are shown as mean ± S.E.M. from three independent experiments.

Supplementary Fig. S7

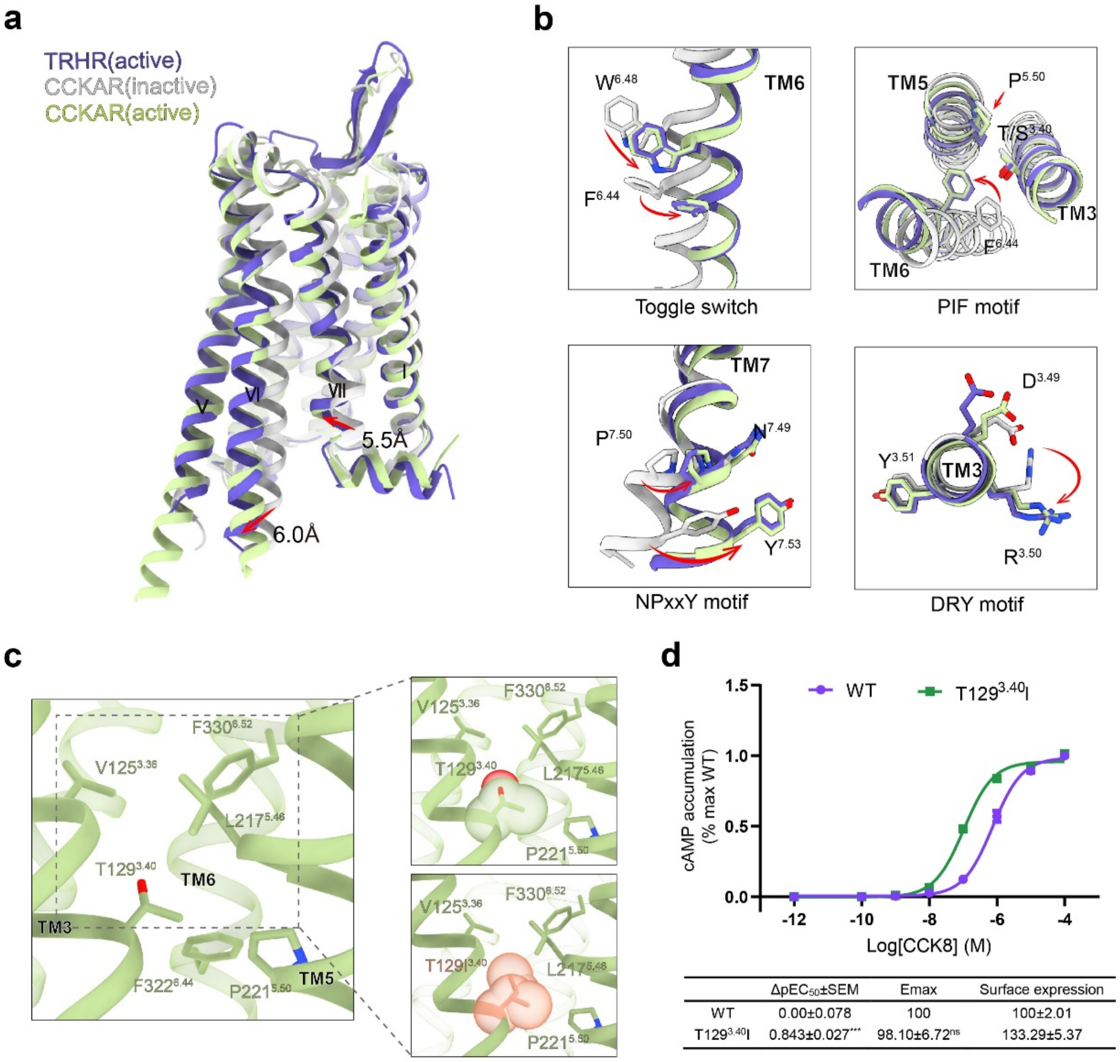

	ΔpEC_{50}±SEM	Emax	Surface expression
WT	0.00±0.078	100	100±2.01
$T129^{3.40}I$	$0.843±0.027^{***}$	$98.10±6.72^{ns}$	133.29±5.37

Fig. S7 Activation mechanism of TRHR.

a Structural comparison of the active TRHR with active (PDB:7EZM) and inactive CCK_AR (PDB:7F8Y). **b** Conformational changes of the conserved "micro-switches" including Toggle switch, PIF, NPxxY and DRY motifs. **c** Close examination of the non-conserved $T129^{3.40}$ in "PIF" motif of CCK_AR. Replacement of $T129^{3.40}$ with $I^{3.40}$ may enhance the hydrophobic interactions with $V125^{3.36}$, $L217^{5.46}$ and $F330^{6.52}$ in CCK_AR. **d** The effect of $T129^{3.40}I$ mutation in CCK_AR on CCK8-induced cAMP accumulation. Data are shown as mean ± S.E.M. from at least three independent experiments performed in technical triplicate. The cell surface expression and E_{max} values were normalized to wild-type CCK_AR. $^{ns}P > 0.05$; $^{***}P < 0.001$ by one-way ANOVA followed by Dunnett's post-test, compared with the response of the WT.

Supplementary Fig. S8

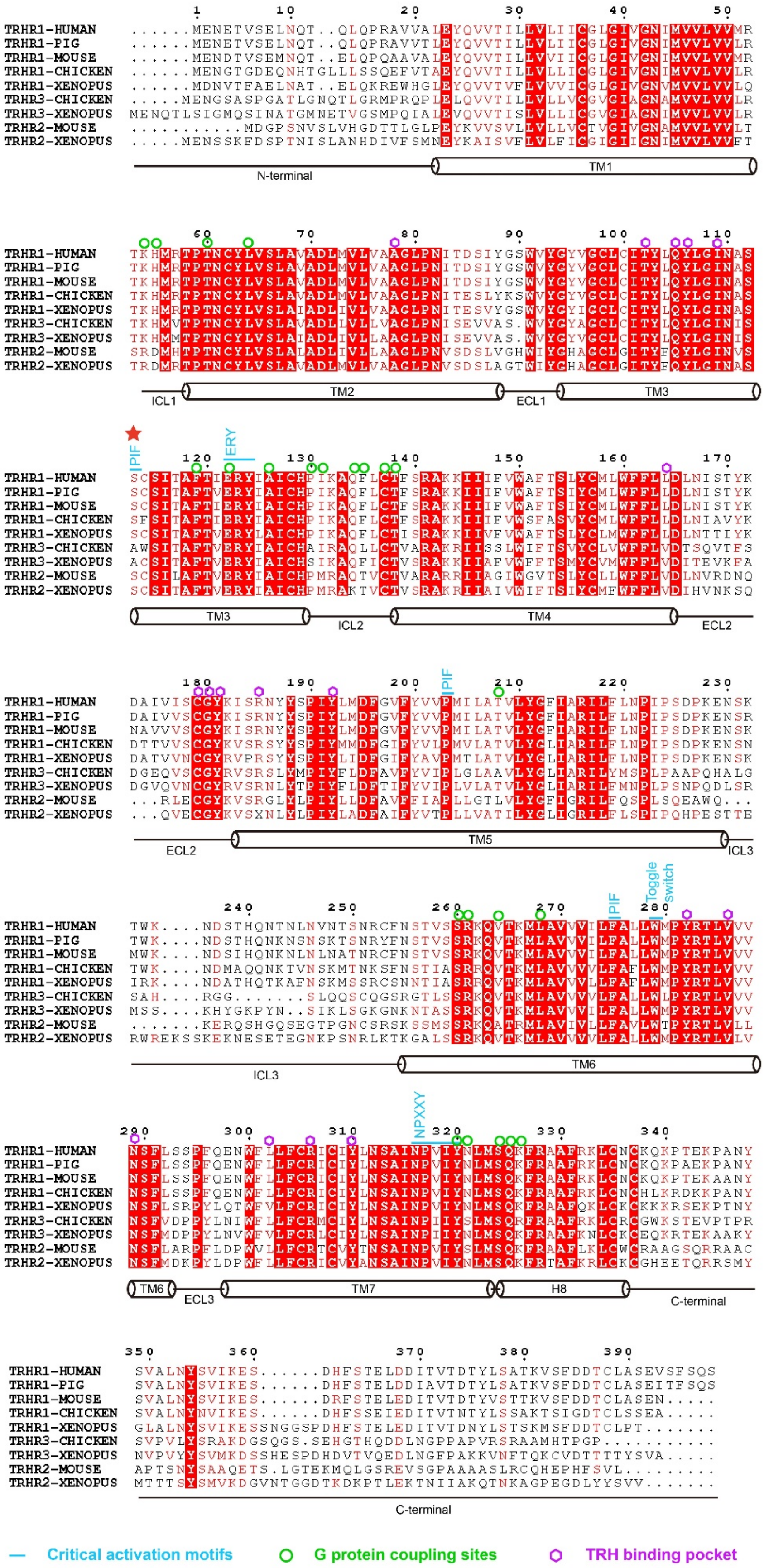

Fig. S8 Sequence alignment of TRH receptors from different species.

Multiple sequence alignment of TRH receptors from Human, Pig, Mouse, Chicken and Xenopus. The critical activation motifs, Gq coupling sites and TRH binding pockets are highlighted. Secondary structure elements are annotated underneath the sequence based on our structure of human TRHR1.

Supplementary Fig. S9

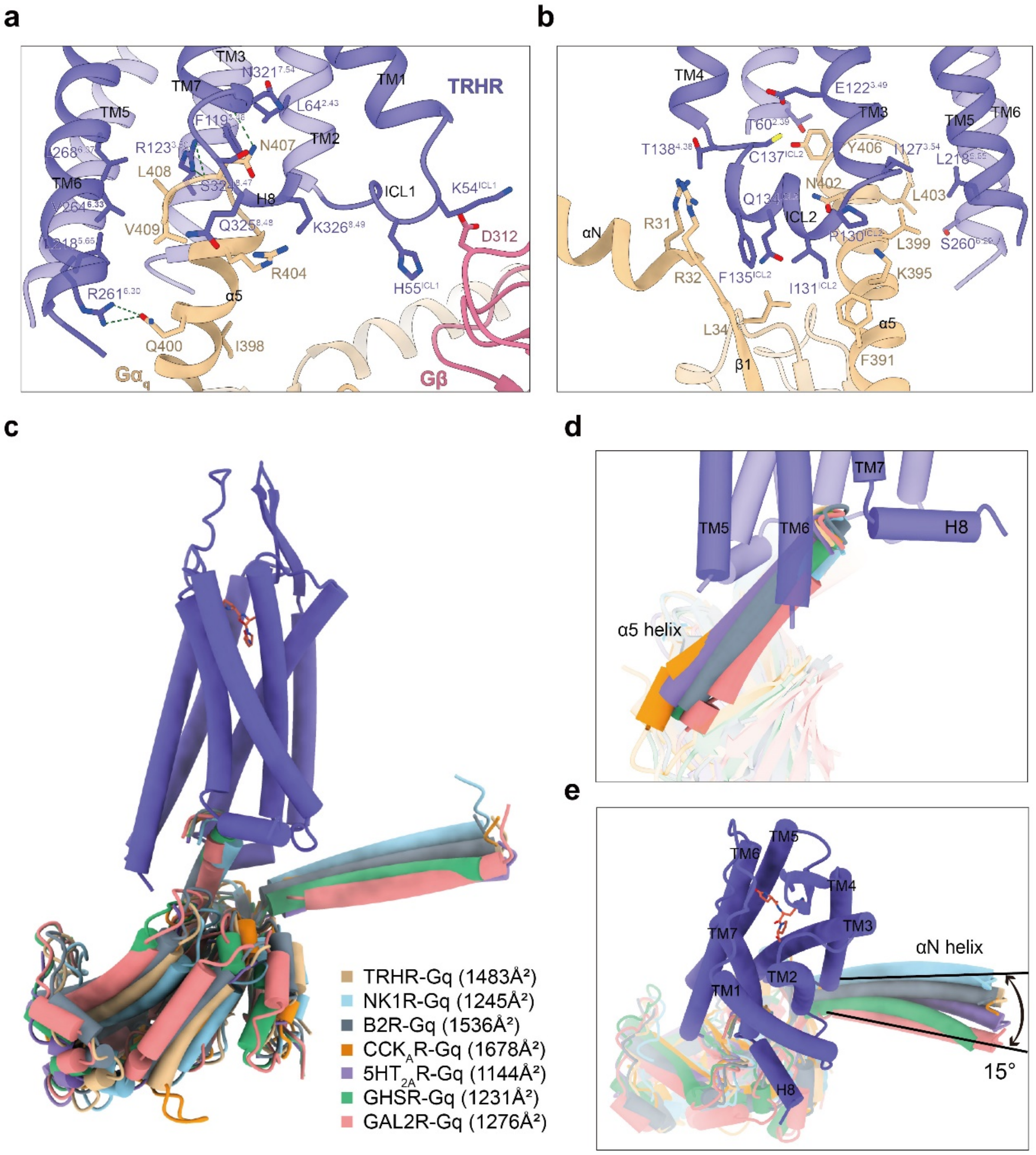

Fig. S9. The Gq coupling mode of TRHR.

a-b Detailed interactions between TRHR (Slate blue) and Gq. Hydrogen bonds are depicted as green dashed lines. **c-e** Structural comparison of the Gq-binding modes between TRHR and other reported GPCRs including neurokinin 1 receptor (NK1R, PDB: 7P00); bradykinin receptor type 2 (B2R, PDB: 7F2O); cholecystokinin A receptor (CCK$_A$R, PDB: 7EZM); serotonin receptor type 2A (5-HT$_{2A}$R, PDB: 6WHA); ghrelin receptor (GHSR, PDB: 7F9Y); galanin receptor 2 (GAL2R, PDB: 7WQ4). Structures were aligned by the receptors; only TRHR was shown for clarity. The GPCR-Gq interface area were measured by Chimera 1.15.

Supplementary Fig. S10

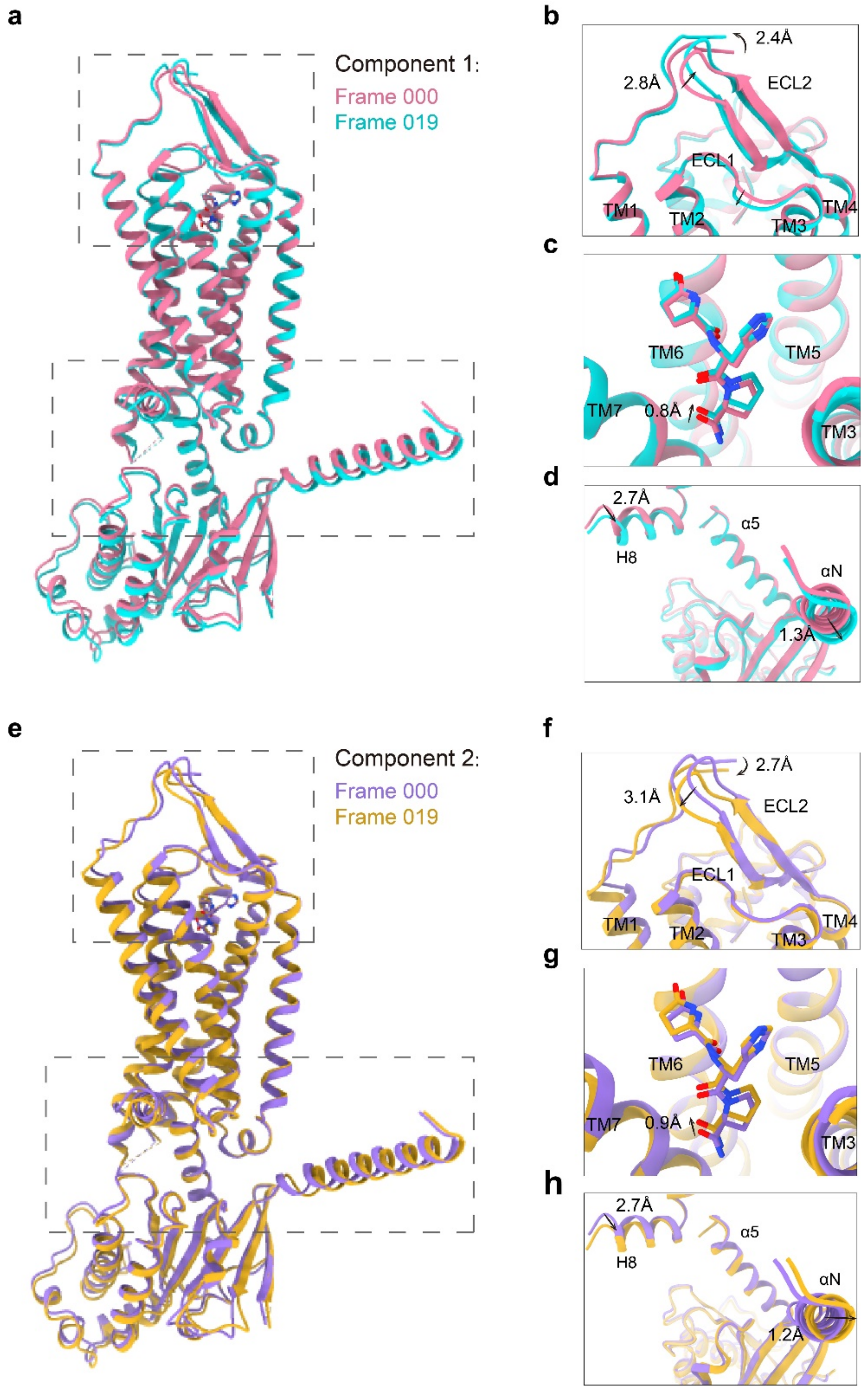

Fig. S10 The TRHR-Gq complex motions from 3D variability analyses.

a-d Superposition of the models built into cryo-EM maps from frame 000 (pink) and frame 019 (blue) of component 1. TRHR motions were measured from the Cα of Q13$^{\text{N-terminal}}$, D173$^{\text{ECL2}}$ and C335$^{8.58}$. Gαq protein motion was measured from Cα of L5$^{\alpha N}$. **e-h** Superposition of the models built into cryo-EM maps from frame 000 (purple) and frame 019 (yellow) of component 2. Receptor motions were measured from the Cα of Q13$^{\text{N-terminal}}$, D173$^{\text{ECL2}}$ and C335$^{8.58}$. Gαq protein motion was measured from the Cα of L5$^{\alpha N}$.

Supplementary Table S1

Cryo-EM data collection, model refinement and validation statistics.

	TRHR-Gq complex
**Data collection and processing**	
Magnification	49,310
Voltage (kV)	300
Electron exposure (e^{-}/Å^2)	64
Defocus range (μm)	-1.0 ~ -2.5
Pixel size (Å)	1.014
Symmetry imposed	C1
Initial particle projections (no.)	3,687,308
Final particle projections (no.)	324,020
Map resolution (Å)	2.7
FSC threshold	0.143
Map resolution range (Å)	2.2-4.0
**Refinement**	
Initial model used	7WQ3, 7WQ4 and AlphaFold2
Model resolution (Å)	2.8
FSC threshold	0.143
Model resolution range (Å)	2.2-4.0
Map sharpening *B* factor (Å^2)	-98.60
Model composition	
Non-hydrogen atoms	8,988
Protein residues	1,143
B factors (Å^2)	
Protein	103.36
Ligand	35.33
R.m.s. deviations	
Bond lengths (Å)	0.005
Bond angles (°)	0.719
Validation	
MolProbity score	1.52
Clashscore	5.64
Rotamer outliers (%)	0.10
Ramachandran plot	
Favored (%)	96.62
Allowed (%)	3.38
Disallowed (%)	0.00

Supplementary Table S2

TRH-induced G-protein dissociation assays of wild-type and mutant TRHR.

	**Sample size**	**ΔpEC$_{50}$±SEM^{a}**	**Emax± SEM^{a} (%WT)**
WT	44	0.00±0.032	100
△N12	3	-0.350±0.123^{ns}	69.77±4.90*
Q13A	6	0.074±0.044^{ns}	108.98±2.30^{ns}
L14A	6	-0.085±0.057^{ns}	94.71±6.23^{ns}
Q15A	6	-0.001±0.027^{ns}	102.57±3.54^{ns}
P16A	6	0.021±0.060^{ns}	91.82±6.89^{ns}
R17A	6	0.099±0.062^{ns}	106.92±3.27^{ns}
△N18	3	0.076±0.076^{ns}	48.07±4.52**
T102$^{3.29}$A	3	-0.622±0.044***	78.75±3.63^{ns}
Q105$^{3.32}$A	3	-0.863±0.051***	79.80±4.87^{ns}
Y106$^{3.33}$A	3	nd	nd
I109$^{3.36}$A	3	-0.519±0.105**	43.10±7.91**
W160$^{4.60}$A	3	nd	nd
L164$^{4.64}$A	3	-0.028±0.046^{ns}	126.06±17.07*
C179^{ECL2}A	3	nd	nd
Y181^{ECL2}A	3	nd	nd
R185$^{5.32}$A	3	-2.169±0.018***	52.04±9.55**
Y192$^{5.39}$A	3	nd	nd
Y282$^{6.51}$A	3	nd	nd
V286$^{6.55}$A	3	-0.542±0.075**	85.14±5.34^{ns}
N289$^{6.58}$A	3	-1.345±0.112***	59.77±5.41*
L302$^{7.35}$A	3	-1.272±0.057***	77.81±10.94*
R306$^{7.39}$A	3	-2.415±0.128***	28.76±3.77***
Y310$^{7.43}$A	3	-0.487±0.025*	109.26±23.48^{ns}
S113$^{3.40}$A	3	-0.106±0.034^{ns}	116.74±5.48^{ns}
S113$^{3.40}$I	3	-1.331±0.115***	75.87±7.45*

[a]Data were analyzed using a three-parameter logistic equation to determine pEC50 and Emax. Emax was normalized to the WT which was set to 100%. All data are shown as mean ± S.E.M. from at least three independent experiments performed in technical triplicate. nd, not determined. $^{ns}P > 0.05$; $*P<0.05$; $**P<0.01$; $***P<0.001$ by one-way ANOVA followed by Dunnett's post-test, compared with the response of the WT.

Supplementary Table S3

Cell surface expression of wild-type and mutant TRHR.

	Sample size	Expression± SEM[a] (% WT)
WT	14	100
△N12	3	106±12
Q13A	6	109±10
L14A	6	84±12
Q15A	6	98±7
P16A	6	103±9
R17A	6	99±12
△N18	3	87±3
$T102^{3.29}A$	3	97±13
$Q105^{3.32}A$	3	44±8
$Y106^{3.33}A$	3	123±15
$I109^{3.36}A$	3	100±6
$W160^{4.60}A$	3	97±11
$L164^{4.64}A$	3	105±12
$C179^{ECL2}A$	3	76±6
$Y181^{ECL2}A$	3	131±12
$R185^{5.32}A$	3	105±11
$Y192^{5.39}A$	3	83±9
$Y282^{6.51}A$	3	91±9
$V286^{6.55}A$	3	57±8
$N289^{6.58}A$	3	60±4
$L302^{7.35}A$	3	125±6
$R306^{7.39}A$	3	45±10
$Y310^{7.43}A$	3	107±17
$S113^{3.40}A$	3	91±9
$S113^{3.40}I$	3	109±16

[a]Data are shown as mean ± S.E.M. from at least three independent experiments performed in technical triplicate.

Supplementary Table S4

Interaction of TRH with TRHR.

TRH	TRHR	Interaction
**pGlu^{1}**	Tyr106$^{3.33}$	Hydrogen bond
	Tyr181^{ECL2}	Polar interaction
	Arg185$^{5.31}$	Hydrogen bond
	Tyr192$^{5.39}$	Polar interaction
	Tyr282$^{6.51}$	Hydrogen bond
	Val286$^{6.55}$	Van der waals force
	Asn289$^{6.58}$	Hydrogen bond
	Leu302$^{7.35}$	Van der waals force
**His^{2}**	Tyr102$^{3.29}$	Hydrogen bond
	Gln105$^{3.32}$	Van der waals force
	Tyr106$^{3.33}$	Hydrogen bond and π-π interaction
	Trp160$^{4.60}$	π-π interaction
	Leu164$^{4.64}$	Van der waals force
	Cys179^{ECL2}	Van der waals force
	Gly180^{ECL2}	Polar interaction
	Tyr181^{ECL2}	π-π interaction
	Tyr282$^{6.51}$	Van der waals force
	Arg306$^{7.39}$	Hydrogen bond
**Pro-NH$_2^{3}$**	Ala78$^{2.57}$	Van der waals force
	Gln105$^{3.32}$	Van der waals force
	Tyr106$^{3.33}$	Hydrophobic interaction
	Ile109$^{3.36}$	Van der waals force
	Tyr282$^{6.51}$	Polar interaction
	Arg306$^{7.39}$	Hydrogen bond
	Tyr310$^{7.43}$	Hydrogen bond
